# An Electronic Patient-Reported Outcome Mobile App for Data Collection in Type A Hemophilia: Design and Usability Study

**DOI:** 10.2196/25071

**Published:** 2021-12-01

**Authors:** Francesco Petracca, Rosaria Tempre, Maria Cucciniello, Oriana Ciani, Elena Pompeo, Luigi Sannino, Valeria Lovato, Giancarlo Castaman, Alessandra Ghirardini, Rosanna Tarricone

**Affiliations:** 1 Centre for Research in Health and Social Care Management (CERGAS) Government, Health and Non Profit Division SDA Bocconi Milan Italy; 2 Roche SpA Monza Italy; 3 University of Edinburgh Business School Edinburgh United Kingdom; 4 Institute of Health Research University of Exeter Medical School Exeter United Kingdom; 5 SODc Malattie Emorragiche e della Coagulazione Azienda Ospedaliero Universitaria Careggi Firenze Italy; 6 Department of Social and Political Sciences Bocconi University Milan Italy

**Keywords:** mobile apps, mHealth, hemophilia A, rare diseases, usability, user-centered design, design science, mobile phone

## Abstract

**Background:**

There is currently limited evidence on the level and intensity of physical activity in individuals with hemophilia A. Mobile technologies can offer a rigorous and reliable alternative to support data collection processes but they are often associated with poor user retention. The lack of longitudinal continuity in their use can be partly attributed to the insufficient consideration of stakeholder inputs in the development process of mobile apps. Several user-centered models have been proposed to guarantee that a thorough knowledge of the end user needs is considered in the development process of mobile apps.

**Objective:**

The aim of this study is to design and validate an electronic patient-reported outcome mobile app that requires sustained active input by individuals during POWER, an observational study that aims at evaluating the relationship between physical activity levels and bleeding in patients with hemophilia A.

**Methods:**

We adopted a user-centered design and engaged several stakeholders in the development and usability testing of this mobile app. During the concept generation and ideation phase, we organized a need-assessment focus group (FG) with patient representatives to elicit specific design requirements for the end users. We then conducted 2 exploratory FGs to seek additional inputs for the app’s improvement and 2 confirmatory FGs to validate the app and test its usability in the field through the mobile health app usability questionnaire.

**Results:**

The findings from the thematic analysis of the need-assessment FG revealed that there was a demand for sense making, for simplification of app functionalities, for maximizing integration, and for minimizing the feeling of external control. Participants involved in the later stages of the design refinement contributed to improving the design further by upgrading the app’s layout and making the experience with the app more efficient through functions such as chatbots and visual feedback on the number of hours a wearable device had been worn, to ensure that the observed data were actually registered. The end users rated the app highly during the quantitative assessment, with an average mobile health app usability questionnaire score of 5.32 (SD 0.66; range 4.44-6.23) and 6.20 (SD 0.43; range 5.72-6.88) out of 7 in the 2 iterative usability testing cycles.

**Conclusions:**

The results of the usability test indicated a high, growing satisfaction with the electronic patient-reported outcome app. The adoption of a thorough user-centered design process using several types of FGs helped maximize the likelihood of sustained retention of the app’s users and made it fit for data collection of relevant outcomes in the observational POWER study. The continuous use of the app and the actual level of engagement will be evaluated during the ongoing trial.

**Trial Registration:**

ClinicalTrials.gov NCT04165135; https://clinicaltrials.gov/ct2/show/NCT04165135

## Introduction

### Background

Patients with hemophilia A, an X-linked recessive bleeding disorder that occurs in approximately 1 in 5000 live male births, suffer from bleeding episodes, especially in their joints and muscles, and moderate impairment of balance and mobility associated with reduced bone mineral density in both adolescence and adulthood [1]. Because of these limitations, patients with hemophilia typically exhibit reduced levels of physical activity [2].

Few empirical studies have reported on the level and intensity of physical activity in small cohorts of children and adolescents [3-5] and in adult populations with hemophilia A [6-9]. These studies were based on either accelerometers or patient-reported questionnaires and showed that the recommended quantity and quality levels of physical activity were often unmet, with the degree of joint damage accounting for only a small fraction of the observed variability [10]. However, no study has measured the physical activity levels in the hemophilia population and evaluated the correlation between the sequelae of different bleeds and the consequent limitations on physical activity levels.

POWER, a multicenter, noninterventional, prospective study aims at contributing to fill this gap by evaluating the relationship between physical activity levels (and intensity) and bleeding in a target population of approximately 150 individuals aged between 12 and 50 years with severe (Factor VIII<1%) or moderate (Factor VIII≥1% and Factor VIII≤2%) hemophilia A without inhibitors against Factor VIII. The study was approved by the Ethics Committee of each participating clinical center and registered at ClinicalTrials.gov (NCT04165135).

This study leverages the widespread availability, low cost, and high degree of reliability of mobile technologies [11] to support the collection of significant amounts of data, including physical activity levels and patient-reported outcome measures (PROMs) [12]. In the field of hemophilia, previous studies have addressed the potential of telehealth-delivered interventions and mobile health (mHealth) solutions to enhance patient adherence to medication and promote independence in disease management [13], improve record keeping [14], and create patient communities that facilitate the interaction of people with hemophilia [15], particularly when they move to adult treatment centers and may report significant feelings of isolation [16]. Although the potential of mHealth technologies to support data collection processes in hemophilia is essentially unexplored, data collection in the POWER study instead relies on 2 different digital devices. The physical activity levels were measured daily in terms of active minutes, the metabolic equivalent of tasks, and the step counts by a wearable fitness device (also called *fitness tracker*) used continuously during the study participation. Concurrently, other relevant self-reported outcome domains (bleeds, medications used for bleeds treatment, health-related quality of life, visual analog scale for pain, etc) were registered through an electronic patient-reported outcome (ePRO) app installed on smartphones or tablets after enrollment.

Although a fitness tracker collects physical activity levels through passive sensing, without requiring any extra effort to input data and with very limited engagement with the device besides the need to wear it, the ePRO app requires sustained active input by study participants.

### Objectives

User engagement and the continuous app use are persisting challenges in mHealth app implementation [17], with poor user retention being observed in the real world [18,19]. This is also true for ePRO systems, whose interfaces should be continuously monitored and improved to reduce the attrition rates in clinical trials and to enhance the retention postimplementation in clinical practice [20]. A decreasing adherence rate to electronic reporting has also been observed in previous studies that involved digital solutions for patients with hemophilia [21-23].

This effect can be partly attributed to the lack of stakeholder input in the development of mHealth technologies: the apps are often made available to the public without sufficient attention devoted to their design [24] and without a thorough understanding of the context of their proposed deployment and the needs of their end users, regardless of whether they are patients, caregivers, or clinicians [25].

Therefore, to maximize electronic outcome reporting and to ensure continuous data collection throughout the POWER study, we incorporated users’ expectations, experiences, and needs in the design process of the ePRO app. This paper reports on the process adopted for the development of a mobile app aimed at collecting PROMs in patients with hemophilia A during the POWER study.

## Methods

### Theoretical Framework: User-Centered Design Approach

We adopted a user-centered design (UCD) and engaged prominent stakeholders in the development of the ePRO app [26]. Among the existing design methods of mHealth apps, UCD primarily focuses on the tasks or activities that the users must accomplish and identifies the corresponding user needs to tackle [27].

According to the UCD approach, during the concept generation phase, a thorough investigation of the needs is conducted to understand the environment of the end users and their requirements. On the basis of this investigation, a set of functional requirements were identified for the ePRO app through thematic analysis, leading to the design of a prototype. The next phases of the design cycle included continuous, iterative evaluation and refinement of the prototype and its usability testing.

### Study Design

**Figure 1:**
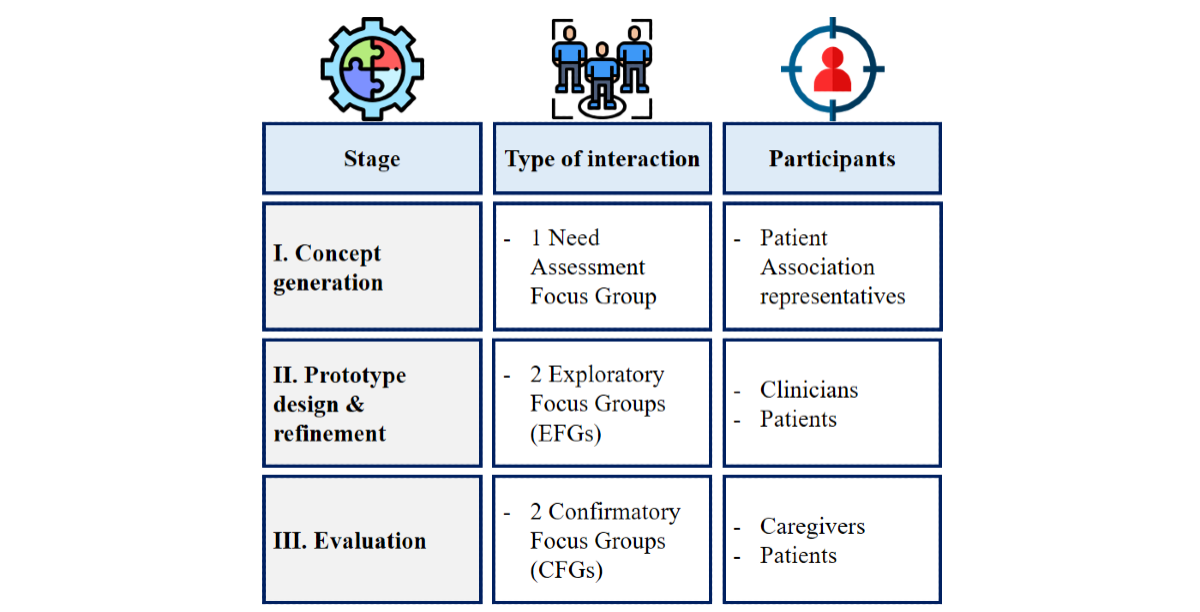
Design process of the electronic patient-reported outcome app.

To improve the app rapidly during the user-centered process, we used focus groups (FGs) as a human factor research technique [28], which could provide appropriate evaluations owing to their flexibility and their ability to probe the participants on key design ideas [29]. Figure 1 shows the various design phases adopted to build the final app.

In harmony with UCD, the initial concept generation phase aimed to analyze the environment of the projected end users of the app and to determine their specific requirements in the context of the POWER study. An initial prototype of the app conceived on the basis of the study goals defined in the protocol was used as a starting point and included 3 main screens: (1) the home page, where recent interactions with the app and activities due were listed; (2) the questionnaire page, through which all the PROMs could be accessed and completed; and (3) the My Health Diary page, where all the recorded data could be retrieved.

The need-assessment FG aimed to elicit specific design requirements and specifications for the prototype app and to bring the environment of the intended end users to focus. The script of this initial meeting revolved around: (1) the definition of functional features to be included in the app to maximize the participants’ engagement with data collection; (2) the suggestion of app characteristics, intended as elements that would qualify the app’s visual appearance and its speed or ease of usage; and (3) the discussion of a specific design feature of mHealth studies: the preference between having the app installed on the participant’s own device (thus following a personal device strategy) or receiving a study device at enrollment with the app downloaded on it.

On the basis of the results of this preliminary activity, we implemented a set of functionalities and engagement strategies and discussed them during the following cycles of the design process, when 2 complementary, yet different sets of FGs were run: exploratory FGs (EFGs) and confirmatory FGs (CFGs) [28].

EFGs were conducted to seek additional inputs for improvement, to refine the prototype, and to maximize the likelihood of technology acceptance. A moderator demonstrated the intended use of the mobile app, while participants were asked to provide their feedback on the proposed prototype during an open discussion.

When the finalized version of the app was completed, the CFGs aimed at gathering evidence of its preliminary efficacy. After a brief introduction of the study objectives, the participants were provided with a smartphone or a tablet equipped with the ePRO mobile app, presented with its use case scenarios, and asked to perform a list of 10 different activities (Textbox 1).

After the test, the participants were asked to quantitatively assess the usability of the app, a multidimensional property associated with attributes such as ease of use, user satisfaction, attractiveness of the layout, and error rates compared with the intended use [30]. According to the World Health Organization, usability testing constitutes the initial step of the evaluation process of any digital health technology [31]. To evaluate the usability of the ePRO app, participants completed the mHealth app usability questionnaire (MAUQ), an 18-item questionnaire recently developed by scholars on a 7-point Likert scale ranging from 1 (*strongly disagree) to 7 (strongly agree)* [32]. The MAUQ is the first scale developed to specifically assess the usability of mHealth apps, and its reliability and validity were shown and compared against other commonly used questionnaires that were not strictly designed for mHealth apps [32]. For the app validation process, we presented the participants with the standalone, patient-specific version of the questionnaire, which included 3 main dimensions: (1) ease of use, (2) interface and satisfaction, and (3) usefulness. According to the questionnaire scoring method, higher average values indicated higher usability levels of the app. The MAUQ is yet to be validated in Italian: we used forward and backward translation to adapt the instrument but did not aim to formally validate it, given the small number of panel components.

**Use case scenarios for the electronic patient-reported outcome app (N=10).**

1. You have just been enrolled in the POWER study. Go to the *Study Details* section to read the main characteristics of the study and the reasons why your participation is significant.
2. Use the *Medication Reminder* function to set an alert every 3 days at 6:30 PM.
3. Report a bleeding episode that happened yesterday in your right thigh and was treated with Factor VIII inhibitors. The bleeding ended and resulted in no days away from school or work.
4. Complete the *Health Questionnaire* you have pending on the home screen. You are feeling great today!
5. Ask the chatbot if your wristband is synchronized with the ePRO mobile device.
6. Fill in the *Assigned Treatment* questionnaire that you find in the *Messages* section.
7. After a follow-up with your clinician, your therapeutic plan has been modified. Modify the *Medication Reminder* settings and set an alert every Tuesday and Thursday at 8:30 AM.
8. The month is coming to an end: fill the *Patient* and *Caregiver Burden* questionnaire and report 2 days away from work in the past month.
9. Go back to read the *Enrollment Update* section where updated statistics on enrolled participants are reported.
10. Check the *My Diary* section to verify that the information you have inputted are available and easily accessible.

### Participants

We invited different target participants to undergo the various rounds of FGs. The initial need-assessment FG was held in February 2019, with the participation of 4 representatives of patient associations for type A hemophilia. The choice to run this initial meeting with representatives of the most established patient associations in Italy was to maximize the understanding of the particular needs of the targeted end user groups, owing to their continuous and long-term contact with a significant number of affected individuals.

Two different stakeholder groups participated in the subsequent EFGs: 4 hematologists in the clinician FG held in May 2019, whereas 5 patients with hemophilia A in the patient EFG in July 2019.

After the finalization of the refined app, we only involved the intended end users during the CFG phase, with patients and their parents (when patients were minor) being asked to ultimately test the app. Prototype validation through usability testing is usually achieved with 2 to 3 cycles of testing [33], whereas for data saturation, small samples of 5 participants typically identify approximately 80% of the usability issues [34,35]. In this study, 2 different meetings were held in November 2019 and January 2020, with 4 and 5 participants, respectively.

Clinicians participating in the EFG were selected among the study centers on a voluntary basis, whereas patients for both EFGs and CFGs were recruited through the patient associations involved in the initial stage of the design process. Health care professionals were eligible if they were hematologists specializing in bleeding disorders and could speak and read Italian. Eligibility criteria for patients included: confirmed diagnosis of hemophilia A, the ability and willingness to complete outcome questionnaires on the ePRO app, the ability to provide their written consent, and the ability to interact in Italian. Parents of underage patients were invited to participate if their children met the clinical eligibility requirements under the study protocol and if they were willing to provide their informed consent and to test the ePRO app. No rewards or compensation were offered to the individuals participating in the study, whereas a participation fee was awarded to the patient associations involved.

### Data Analysis

All FGs occurred in a conference room, lasted for approximately 90 minutes, and were recorded (audio only) and then professionally transcribed. We obtained the written informed consent from all the participants before the start of the meeting. The participants were asked to provide essential personal data, including their age, their professional role (if applicable), and their self-reported comfort in the use of mobile devices. During FGs, we used sticky notes to collect the recommendations of the participants regarding design changes and content review. The analysis of the need-assessment and exploratory FGs was facilitated by Dedoose qualitative software (SocioCultural Research Consultants), a web-based platform for mixed methods analysis [36] that enabled the identification of recurrent themes and the development of a coherent coding index. An inductive analysis was performed by 2 researchers (FP and MC), with emerging themes identified by the systematic reading and coding of the transcripts. Different opinions between the coders were discussed by the research team to reach a consensus.

## Results

### Participant Characteristics

Of the 22 participants, 11 (50%) were patients, 4 (18%) were patient representatives, 4 (18%) clinicians, and 3 (14%) parents of young patients. Detailed information about the category, age, and gender of the study participants by each research stage has been presented in Table 1.

**Table 1:** Participant characteristics (N=22).

Characteristics		Need-assessment FG^a^, n (%)	EFGs^b^, n (%)	CFGs^c^, n (%)	Total, n (%)
**Category**					
	Caregiver	0 (0)	0 (0)	3 (33)	3 (14)
	Clinician	0 (0)	4 (44)	0 (0)	4 (18)
	Patient	0 (0)	5 (56)	6 (67)	11 (50)
	Patient representative	4 (100)	0 (0)	0 (0)	4 (18)
**Age (years)**					
	<30	0 (0)	4 (44)	4 (44)	8 (36)
	30-39	0 (0)	1 (11)	1 (11)	2 (9)
	40-49	1 (25)	0 (0)	1 (11)	2 (9)
	50-59	1 (25)	2 (22)	1 (11)	4 (18)
	>60	2 (50)	2 (22)	2 (22)	6 (27)
**Gender**					
	Male	3 (75)	6 (67)	7 (78)	16 (73)
	Female	1 (25)	3 (33)	2 (22)	6 (27)

^a^FG: focus group.

^b^EFG: exploratory focus group.

^c^CFG: confirmatory focus group.

### Concept Generation: Need-Assessment FG

Thematic analysis of verbatim transcripts of the need-assessment FG led to the identification of 6 themes that were of help in refining the initial prototype.

First, the participants stated that it was paramount to guarantee maximum simplification of the app functionalities, ensuring that the burden on individuals was minimized to what was strictly necessary for study purposes. One patient representative said the following:

Rather than improving the quality and quantity of the app experience, I believe it is fundamental to make it easy, simple, user-friendly, and self-explanatory, making it possible to access it also for somebody who would not want to know how it actually works.

Patient Representative, 53 years

Second, the participants made specific recommendations about the sense-making process related to the app, which should leverage the intrinsic motivation of individuals to be part of a noteworthy initiative where the individual benefit is negligible when compared with the profit for the entire community of interest. Therefore, numerous patient representatives suggested the inclusion of specific functionalities to give feedback on the study progress and make every individual part of a larger community. For example, one patient representative vigorously emphasized this point, as follows:

The appeal and the willingness to participate and continuously input data are based on the fact that somebody believes in what the study is proposing, everything else is just a plus...you must necessarily find a way to transfer the sense of what you are asking people to do.

Patient Representative, 64 years

Third, the FG participants suggested that the app should ensure maximum integration, refraining from any request for data or for doing tasks that can already be performed through different means.

Fourth, a common theme pertained to the need to limit the feeling of external control that a digital solution could exercise on individuals, which can ultimately be linked back to the need to ensure that the participants were reassured about the study’s purposes and modalities. For instance, one participant said the following:

No one should ever get the feeling of being controlled by the app or somebody behind it. I have no need for further sources of control that can make me feel more ill than I already am.

Patient representative, 53 years

The aesthetics of the user interface were identified as another factor to improve, with the FG participants advising to adopt different color sets to make the layout catchier, but not trivial, than the initial one and to enable individuals to tailor some of the app’s graphical features to their personal preferences.

One final emerging theme pertained to the type of device used during the POWER study. The participants unanimously agreed that providing them with an additional device exclusively for study purposes would prove excessively burdensome and would not help maximize patient engagement, probably generating increased attrition rates instead. One patient representative stated as follows:

Honestly speaking, to propose in 2019 to participate in a study in which individuals are obliged to use an additional device could be detrimental to the overall study participation.

Patient Representative, 40 years

### Changes to the App After Need-Assessment

Consistent with the demands expressed during the preliminary phase and coded through qualitative analysis, the app underwent the following modifications and additions:

- *The Study details* and *Enrollment update* pages were added to provide the participants with valuable information about the study and its status, leveraging the required sense-making process;
- Environmental alerts were added to prompt the participants to input the required outcome data;
- Users were given the opportunity to tailor the design features of the app based on their preferences;
- Acknowledgments on activity completion were reinforced to maximize efficiency by providing visual confirmation that the inputted data had been registered;
- A *Treatment reminder* functionality was included in the app and made accessible on a voluntary basis.

### Prototype Refinement: EFGs

During the 2 EFGs, the clinicians and patients with hemophilia were asked to provide feedback on the refined prototype version of the app to achieve rapid incremental improvements.

During the clinician EFG, the participants univocally acknowledged the significant contribution of the POWER study to provide up-to-date information on the population of interest. Four main codes were identified during the discussion and considered as recommendations to further improve the app.

First, participants suggested avoiding duplications, as some of the sections were potentially overlapping and were not necessarily mutually exclusive. This had primarily related to the structure of the *Home* page, which was initially designed as a repository of the most recent information included in all the other sections of the app.

Second, a recurrent theme pertained to the graphical interface of the app, against which clinicians suggested facilitating the detection of different domains with corresponding bright colors. One clinician explained the following:

I find the app look slightly monotonous...to better highlight the various items and domains, different colors could be used across the different sections of the app.

Clinician, 62 years

Additional recommendations focused on the study update section. The *Study details* and *Enrollment update* sections that were added to the app after the need-assessment phase were appreciated but considered not adequately positioned within the app for accessibility.

Finally, a fourth emerging theme pertained to the possible uses of the app outside of the study settings. To further reduce the patient burden in completing the questionnaires, clinicians suggested machine-readable formats, voice learning, and voice recording. However, these proposals were not technically feasible given the timeframe of the POWER study and, therefore, could not be embedded in the current version of the app.

During the patient EFG that followed, the participants were presented with a refined version of the app that incorporated the main comments collected during the clinician EFG in terms of outlook and content organization.

The coding analysis highlighted several recurrent topics that were brought to attention during the meeting.

First, patients reported their need for support in daily management and coping, expressing a strong willingness to feel a tighter bond with their clinicians and the hope that this could be channeled through enhanced data sharing via the app. In the context of persisting issues in finding appropriate and continuous type of support to sustain patients with hemophilia A daily, there was wide acknowledgment of the potential of digital technologies in closing the existing gap with health care professionals. One patient described this as follows:

To have somebody to actually follow us through our daily struggle and provide us with prompt feedback would mean a lot...sometimes you just have the impression that you get a pre-set response hours later your request.

Patient, 18 years

As a second element, the participants confirmed the need to be supported and facilitated in their participation in the study and in the use of the app to relieve the burden linked to data entry that could cause disaffection with the app, if it were not well supported.

Regarding the *interface design,* the participants only suggested minor revisions in the layout of some questionnaires, particularly to make the reporting of bleeding events more straightforward. Furthermore, one user recommended substituting the human body where bleeds were indicated to make it less stylized and more realistic.

**Figure 2:**
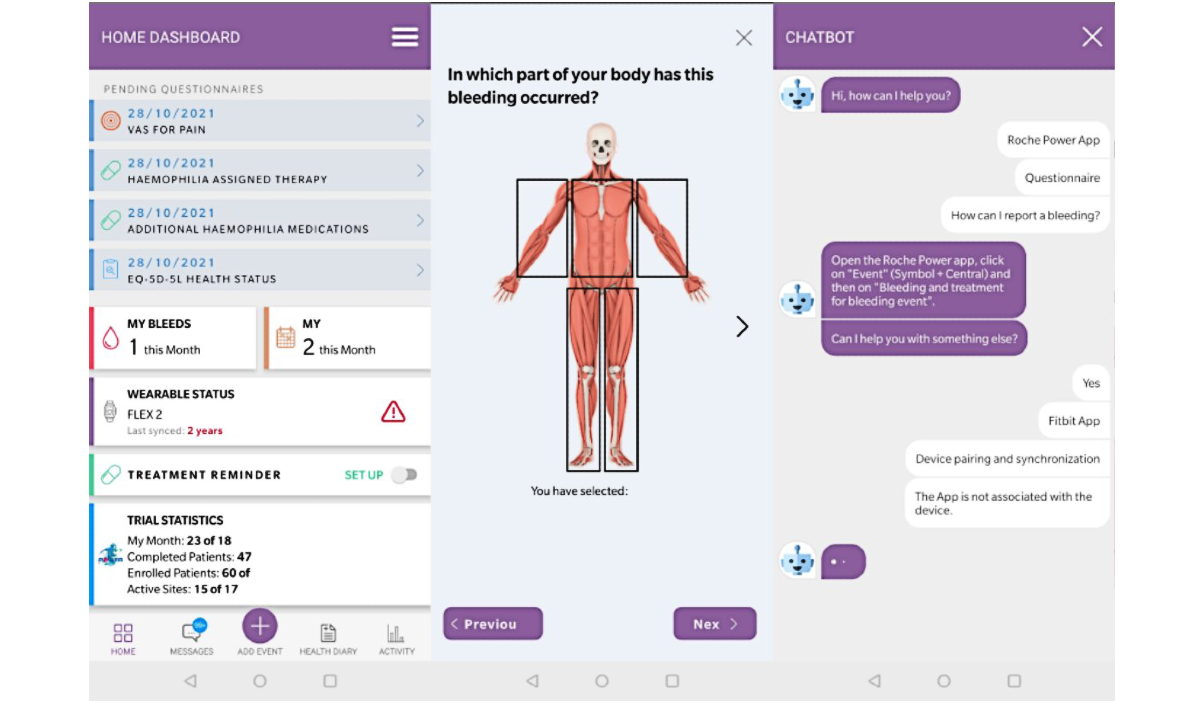
Screenshots of the electronic patient-reported outcome app final prototype.

On the basis of the inputs collected during the EFGs, additional graphical refinements were introduced to further streamline participation in the study: (1) a new chatbot was included to support individuals in finding information related to the app and its functionalities; (2) an additional screen with visual feedback on the number of hours the wearable device had been worn was added to ensure the per-protocol minimum (10 hours/day) was reached and that the observed data were actually registered. The final display of the app home page, chatbot functionality, and bleeding reporting have been shown in Figure 2.

### Usability Evaluation

During the CFGs, participants tested the final version of the app, followed a list of 10 use case scenarios (Textbox 1), and completed the MAUQ to assess their satisfaction with the system usability.

The average scores showed an upward trend: the overall MAUQ score increased from 5.32 (range 4.44-6.23; SD 0.66) to 6.20 (range 5.72-6.88; SD 0.43) over the 2 CFGs, showing increasingly positive feedback on the app usability as its design was further refined.Considerable improvements were observed especially in terms of perceived usefulness, which increased from 4.80 (SD 0.92) to 6.24 (SD 0.40), and the system information arrangement of the app (from 5.30 to 6.17).

During the first CFG, participants reported poor responsiveness of certain features and a few technical problems, primarily when filling some of the PROMs or when using the chatbot. These issues ultimately affected the reported usefulness and the interface evaluation of the app. The technical difficulties were addressed between the 2 meetings, and the app’s usability ratings improved accordingly. During both the meetings, all participants reported being intuitively able to launch the app and requiring minimal support from the study team during the testing.

On the basis of the inputs provided by the CFG participants through qualitative comments, minor additional changes were included in the finalized version of the app that is currently being used in the POWER study.

## Discussion

### Principal Findings

We applied UCD for the development of the ePRO app currently in use in the POWER observational study, adopting an iterative process in which progressive modifications were informed not only by the participants’ inputs but also by the technical feasibility of the proposals. This approach has been identified as particularly effective when adapted to mHealth apps [37], with different frameworks being used as powerful alternatives to the shared design based on the end users’ preferences [27]. A recent integrative review analyzed studies that employed a qualitative methodology for the design, development and testing of mHealth apps, identifying 69 articles, and proposing an integrated methodology structured in 4 different sessions [38]. These results confirm the continuous growth in the literature on user-centered approaches for app development. However, all the design studies included in the review aimed to develop apps to support individuals in actively managing their disease through the adoption of behavior change techniques [39] and other cognitive and emotional strategies.

In contrast, none of the studies intended to support the design of data collection apps, with the exception of a single article that focused on the development process of an app for conducting population surveys, but it was meant to be used by health care professionals only [40].

The design process of the ePRO app for the POWER study is a novel attempt to adopt participatory approaches (and UCD, more specifically) to support the collection of patient-reported data. Although we did not aim to elicit improved self-management behaviors, maximized technology acceptance is a fundamental prerequisite to increase the continuity in data entry and the likelihood of a study’s success. As the precise aims that the app had to pursue were already explicit in the POWER study protocol (ie, to maximize the likelihood of collecting robust outcome data), the design process did not start from scratch and revolved around the *how* rather than the *what*.

Despite this difference in the ultimate aim of the app, some of the adopted design strategies we used are comparable with recurring themes in previous studies.

To sustain user engagement, several of the included features and strategies aimed at powering the intrinsic motivation of the participants, defined elsewhere as altruistic motivation [41]. This is coherent with the belief that although strategies that rely on extrinsic motivation only (*doing something that can lead to an identifiable outcome*) can be effective in the short term, when individuals are intrinsically motivated (*doing something which is felt inherently enriching*), they tend to achieve better results and guarantee their continued participation in the long run [42]. As a result, increased attention was given to the *Study details* and the *Enrollment update* sections, that intend to make the individuals feel they were part of a community and of a mutual effort, which was certified by the growing number of active sites and study participants enrolled.

Concurrently, we included multiple strategies aimed at making the individual experience with the app as efficient as possible, such as the acknowledgment notification to give visual confirmation of the recording of completed questionnaires and the chatbot function to facilitate users in the management of any technical or content-related issues. Furthermore, the questionnaires were tested and revised multiple times to streamline their completion and to minimize the participants’ burden.

Third, to further facilitate the engagement activation process, we tried to take on the challenge of personalization, realizing that technologies should focus on each individual as unique, even in their communication preferences and approaches to data collection processes [43]. Personalization is a recurring theme in app design studies but is typically implemented for ad hoc self-management functionalities or goal setting [44-47]. Instead, given the need to collect the same outcome data for all participants in the POWER study, with no room for content personalization, we included the possibility for users to tailor some of the app graphics and added individual functionalities, such as the treatment reminder, which could be accessed on a voluntary basis. These enhancements do not have a potential interventional effect on the outcomes of interest, and may help generate a greater, personalized bond between the individual user and the mobile app.

In addition to the app content, another domain that might sustain user engagement pertains to the type of mobile device used during the study (either a personal or a study device). Although this ambivalence is a specific feature of mHealth studies, very few studies have attempted to compare the 2 strategies empirically in terms of adherence, with inconclusive statistical results [48]. Despite this debate, there is still no unambiguous settlement on the use of personal devices. However, this strategy, which also goes by the name *Bring Your Own Device*, has been unquestionably identified as the preferable solution during this app’s development process. This option is not exempt from potential shortcomings associated with the need to develop apps that are compatible with a wide range of systems and security settings [49], the potential selection bias that excludes population segments who do not own a smartphone [50], and the impossibility of locking down the device and maximizing the methodological accuracy. Nevertheless, a personal device presents one major advantage that counterbalances all of the previous shortcomings, as it enhances the convenience for patients, potentially reducing their burden and adopting a pragmatic, real-world approach that replicates a setting more familiar to all the participants.

Finally, to enhance the engagement with the ePRO app, we strengthened the connection with the fitness tracker that records physical activity levels. Although wearables are standard technologies that do not allow for discretion in their design and were previously perceived as highly acceptable by patients with severe hemophilia [7], we reinforced their linkage with the ePRO app by including an ad hoc screen that provided participants with feedback on the number of daily hours the tracker has been actually worn and confirmed whether the per-protocol minimum for physical activity levels to be actually registered (10 hours) was being met.

In addition, the ePRO app may indirectly activate engagement by improving communication with the providers using previously recorded data during routine consultations. The need to be more closely monitored by their physicians was reported by the patients during the FGs and has also emerged in previous app design studies [51,52]. Furthermore, the enhancement of communication links between the health care professionals and the patients, as well as the capture of patient-reported data, are considered among the 4 primary ways through which mHealth can improve the management of hemophilia A [14].

By the end of the design process, we achieved broad agreement that the app was easy to use and had an appealing layout, both preconditions for its sustained use over time. As shown previously by other studies [37], 2 iterative cycles of usability testing were sufficient to reach a satisfactory consensus among the participants and the potential end users. The comparisons of usability with other mobile apps that underwent a thorough development processes are difficult to make because this app is not directly aimed at self-management, and this study was among the first to adopt the MAUQ.

### Strengths and Limitations

This research was based on a large sample of participants and included various perspectives by considering the views and needs of the patients, their parents, their representatives, and those of the clinicians during the development process.

Moreover, this study emphasized that user-centered approaches can be applicable, and possibly even more significant, to the development of digital solutions for populations affected with rare diseases, which, by definition, require unique considerations that may be less well-known compared with those for other chronic disease populations. At the same time, the application of the selected methodology to a rare disease group generated additional challenges linked to the difficulties in selecting and enrolling the participants. Working with smaller sample sizes in rare study populations may be the only way to study them, particularly for methodologies that require the physical presence of participants [53], but several limitations should be acknowledged. First, in terms of patient selection, although we always targeted 5 participants in each of the FGs, in a couple of cases, one of the intended participants communicated their unavailability at short notice because of hemophilia-related issues. Furthermore, to facilitate the identification of the potential participants, we not only adhered to the inclusion criteria identified for the POWER study, but also invited patients with hemophilia A with different severity and inhibitor levels. Although some of the participants were not within the target population of the study, all patients with hemophilia A were subject to similar outcome data collection in standard clinical practice and were, thus, entitled to bring their contribution to the development of the app.

Second, the number of practitioners involved was relatively small and their points of view may not be representative of the entire clinician population or of the organizations they represented.

An additional limitation pertains to usability, which was only evaluated before the field testing of the app in the study and was not assessed longitudinally. Usability evaluation should be a continuous process and should not be limited before dissemination in the field. Additional usability evaluations were planned during the study and at the end of the study through ad hoc use metrics aimed at analyzing the use trajectories. Moreover, we based our usability assessment only on the end user testing and did not properly include any heuristic evaluation involving computer scientists, nor assessed the time to task completion. The mobile app was subject to continuous technical evaluations, and the technical development went hand in hand with the content design.

Finally, the UCD process we adopted was mainly based on user and clinical expert involvement, which are however just 2 of the developmental factors could be included in the design phase of an app [54]. We have noted that alternative methodologies have been developed and they are becoming popular in studies on mobile devices, such as the experience sampling method [55].

### Conclusions

The ePRO app will serve as a data collection platform in the POWER observational study. Because all the outcome data collected by the app are directly inputted by the patients, UCD supported the identification of user requirements and the refinement of the app. This process will hopefully meet the users’ expectations and maximize their continuous use of the app throughout the study. The actual level of engagement will be properly monitored during the ongoing POWER study, whereas future research results will assess the effectiveness of this app and demonstrate the value of the development process described here.

## Abbreviations

CFG: confirmatory focus group
EFG: exploratory focus group
ePRO: electronic patient-reported outcome
FG: focus group
MAUQ: mHealth app usability questionnaire
mHealth: mobile health
PROM: patient-reported outcome measure
UCD: user-centered design
